# Bilateral Ureteral Lithiasis and Postrenal Acute Kidney Injury: A Rare Complication of Diabetic Ketoacidosis in a Child

**DOI:** 10.1155/crpe/2711257

**Published:** 2025-08-15

**Authors:** Fabio Rotondo, Sofia Siena, Maria Rosa Pastore, Pasquale Pio Maccarone, Morena Luce Mansueto, Irene Rutigliano

**Affiliations:** ^1^Department of Pediatrics, University Hospital of Foggia, Foggia, Italy; ^2^Department of Pediatrics, IRCCS “Casa Sollievo Della Sofferenza” Hospital of San Giovanni Rotondo, San Giovanni Rotondo, Italy

**Keywords:** acute kidney injury, bilateral stones, diabetic ketoacidosis, kidney stones

## Abstract

Diabetic ketoacidosis (DKA) is a common initial presentation of type 1 diabetes mellitus (T1DM) in children occurring in up to 40% of cases. DKA can also be associated with severe complications, including nephrolithiasis. We present the case of a 12 years and 8-month-old boy who developed acute kidney injury (AKI) secondary to bilateral urinary lithiasis during the onset of T1DM with DKA. After conventional treatment for DKA, laboratory tests showed increased creatinine and azotemia. 24 hours later, he developed lumbar pain and anuria. Plain radiography, ultrasonography, and computed tomography (CT) revealed bilateral renal calculi and pelvic dilation. An urgent bilateral ascending pyelography with stent placement was performed. Right ureteral stenting was successful, but left stenting failed due to an intramural ureteral anatomical variant; thus, a nephrostomy was performed. Diuresis resumed immediately, and renal function normalized over the following days without permanent impairment. To our knowledge, this is the first reported case of bilateral lithiasis with ureteral stenosis in a pediatric patient with DKA. In patients with severe DKA, we recommend routine monitoring of kidney function with a low threshold for CT imaging whenever there is an unexpected rise in creatinine, oliguria/anuria, or lumbar pain. Early multidisciplinary intervention can promptly relieve postrenal obstruction, prevent permanent renal damage, and improve outcomes.

**Summary**

• Bilateral ureteral obstruction from lithiasis is a rare complication of DKA that can lead to AKI and permanent kidney damage.
• In patients with DKA, it is crucial to monitor kidney function for preventing consequences of AKI.

## 1. Introduction

Type 1 diabetes mellitus (T1DM) is a chronic condition that commonly affects children and is caused by the autoimmune destruction of pancreatic beta cells, resulting in insulin deficiency. Although it primarily manifests during childhood, up to one-quarter of cases are diagnosed in adulthood [1, 2]. Clinically, T1DM can present in three ways: the classic presentation includes polyuria, polydipsia, polyphagia, weight loss, with laboratory findings of hyperglycemia, ketonemia, and/or ketonuria; diabetic ketoacidosis (DKA); asymptomatic onset: an incidental finding of hyperglycemia or metabolic abnormalities [3].

DKA is the second most common presentation of T1DM, with an estimated incidence of 30%–40%. Adolescents or individuals from disadvantaged socioeconomic backgrounds are more likely to presentwith DKA [4].

DKA results from a deficiency in circulating insulin levels and increased levels of counterregulatory hormones. These include catecholamines, glucagon, cortisol, and growth hormone. These hormones promote glycogenolysis and gluconeogenesis, leading to hyperglycemia, polyuria, and intravascular dehydration. Simultaneous lipolysis, with the release and oxidation of free fatty acids, facilitates gluconeogenesis, ketogenesis, and the consequent metabolic acidosis [5]. DKA presents symptoms similar to the classic presentation of T1DM but in a more severe form. These include hyperglycemia, ketonemia, and/or ketonuria associated with abdominal pain, anorexia, nausea, vomiting, altered mental status, Kussmaul breathing, and neurological disturbances. The diagnostic criteria for DKA include hyperglycemia: > 200 mg/dL; metabolic acidosis: pH < 7.3 and bicarbonate < 18 mmol/L; ketonemia: beta hydroxybutyrate > 3 mmol/L, ketonuria.

The severity of DKA can be classified as mild, moderate, or severe based on metabolic parameters and clinical presentation [6]. DKA has been associated with several potentially serious complications affecting various organs and systems. Among these, cerebral edema is one of the most feared in pediatric patients. Other complications include electrolyte disturbances (e.g., hypokalemia or hyperkalemia), cardiac arrhythmias, and, more rarely, pancreatitis [7]. A high frequency of acute kidney injury (AKI) in children with DKA (approximately 50%) has been documented [8, 9]. Although AKI is generally attributed to severe dehydration or renal hypoperfusion that leads to intrinsic tubular injury [10], urinary tract obstruction as a cause of AKI is rare, particularly in pediatric patients. The loss of water and electrolytes due to osmotic diuresis secondary to hyperglycemia leads to dehydration [5] which increases urinary calcium and phosphate excretion. Concurrently metabolic acidosis lowers citrate levels and promotes crystallization thereby raising the risk ofcalcium stone formation [11, 12]. It is therefore hypothesized that DKA-induced severe dehydration, hyperglycemia, hyperglycosuria, and acidosis may predispose patients with diabetes mellitus to urinary stone formation [13, 14]. The mechanism of postobstructive AKI is well known. Ureteral obstruction produces increased intratubular pressure, followed by renal vasoconstriction and a rapid decrease in renal blood flow and GFR [15]. If the obstruction is prolonged, interstitial fibrosis and nephron decay starts eventually leading to some degree of chronic kidney disease [16].

## 2. The Case

**Figure 1:**
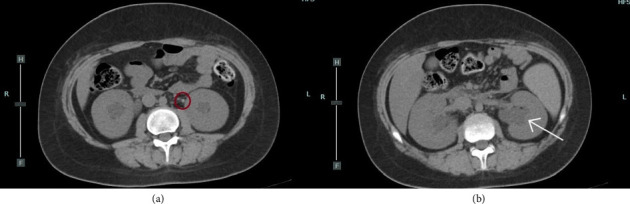
(a) CT scan. The circle indicates the stone located in the left ureter. (b) CT scan. The arrow indicates the dilation of the left pelvi.

**Figure 2:**
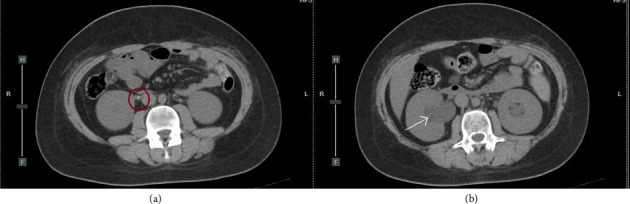
(a) CT scan. The circle indicates the stone located in the right ureter. (b) CT scan. The arrow indicates the dilation of the right pelvi.

A 12-year- and 8-month-old boy with no other known comorbidities was referred to a Hospital in Apulia (Italy) for polyuria, polydipsia, polyphagia, asthenia, and weight loss of approximately 10 kg over the previous month. On admission, the patient had severe dehydration and Kussmaul breathing pattern. Arterial blood gas analysis revealed pH of 7.12, HCO_3_^−^ of 2 mmol/L, blood glucose of 536 mg/dL, corrected sodium (Na^+^) of 137 mmol/L, and corrected potassium (K^+^) of 2.8 mmol/L. Due to the state of severe metabolic acidosis, he was treated with intravenous rehydration and insulin, according to the Italian Society of Pediatrics Endocrinology and Diabetology (ISPED) 2015 DKA protocol [17]. Basal–bolus insulin regimen started, according to protocol, on Day 3 from the onset. On Day 4 from the onset, the boy was transferred to our hospital for further diagnostic evaluation and education on therapeutic management. On admission, his mental status was alert, and vital signs included a blood pressure of 120/80 mmHg, 100 bpm, and body temperature of 36.5°C. His body weight and height were 69 kg (90th–97th percentile) and 156.5 cm (50th–75th percentile), respectively. Lips, oral mucosae, and tongue appeared normal in color and moisture. No lesions, ulcers, or signs of infection were found. His abdomen had striae rubrae on the flanks; it was globular due to adipose tissue, and soft and distended but painful to deep palpation in the mesogastrium and left hypochondrium; peristalsis was normal. His laboratory findings revealed the following results: venous blood gas with a pH of 7.33, HCO_3_^−^ 15.8 mmol/L, ketonemia 1.8 mmol/L, blood urea nitrogen (BUN) 11 mg/dL, creatinine 0.8 mg/dL, glucose 359 mg/dL, HbA1c 10.8%, and C-peptide 0.49 ng/mL. Other laboratory findings included the following results: hematocrit 36.4%, hemoglobin level of 13.4 g/dL, white blood cell count of 6880/μL, platelets count of 157,000/μL, total cholesterol level of 95 mg/dL, high-density lipoprotein (HDL) level of 44 mg/dL, triglycerides level of 110 mg/dL, low-density lipoprotein (LDL) level of 39 mg/dL, aspartate aminotransferase level of 14 U/L, alanine aminotransferase level of 16 U/L, amylase level of 32 U/L, lipase level of 139 U/L, sodium level of 135 mmol/L, potassium level of 2.9 mmol/L, and chloride level of 106 mmol/L. On Day 5 from onset, follow‐up blood tests showed a BUN of 32 mg/dL and creatinine plasma level of 2.2 mg/dL. The patient was diagnosed with having autoantibody-positive T1DM (glutamic acid decarboxylase values of 248 U/mL and positive value over 10 U/mL). The following day, the patient reported lumbar pain and, after a while, anuria; repeated laboratory tests revealed BUN 59 mg/dL and creatinine 5 mg/dL. We performed an abdominal ultrasound examination which showed kidneys in their anatomical position with normal dimensions, morphology, and echotexture; however, a bilateral kidney pelvis ectasia was observed with an anteroposterior diameter of approximately 12 mm on the right and about 9 mm on the left and a poorly filled bladder with thickened walls. An urgent abdominal radiography documented abundant fecal residues but could not clearly identify opacities along the ureters. Therefore, a noncontrast abdominal CT scan was performed revealing a calcification distal to the left pyeloureteral junction and an anteroposterior dilatation of the renal pelvis of approximately 20 mm (Figure 1); similarly, along the proximal segment of the right ureter, in a position slightly more caudal than the contralateral side, another elongated and irregular lithiasic calcification was seen, extending longitudinally for about 1.2 cm with evidence of ureterohydronephrosis and an anteroposterior dilation of the renal pelvis of approximately 23 mm (Figure 2). The renal parenchyma appeared moderately enlarged with edema. The diagnosis was AKI secondary to bilateral lithiasis. The patient was urgently transferred to the Department of Urology for bilateral ascending pyelography with stent placement. The procedure was successfully performed on the right ureter; the ureteral catheter could not be advanced more than 1 cm from the left ureteral orifice, despite repeated attempts, due to a deviation of the ureter's bladder intramural tract (reversed-hook shape). A percutaneous posterolateral left nephrostomy was performed, and diuresis was immediately re-established. During the procedure, the patient developed metabolic acidosis, and he was transferred to the ICU for monitoring and management in a controlled setting. Once the metabolic acidosis was resolved, he returned to the pediatric unit (day 9 from onset) for further clinical and laboratory monitoring. Laboratory tests showed decreasing BUN and creatinine. 24 h urine collection was performed, revealing levels of creatinine of 0.97 g/24 h, uric acid 622 mg/24 h, magnesium 39.1 mg/24 h, citrate 0.74 mmol/24 h, cystine < 0.8 mmol/24 h, cystine/creatinine 90.4 mmol/mol, proteins 1586.1 mg/24 h, oxalate 7.5 mg/24 h, and calciuria 11.8 mg/dL. A follow‐up ultrasound was performed exclusively in the upright position due to intense pain in supine position; it showed normal kidney dimensions and morphology with no dilation of the renal pelvis which appeared to be hypotonic. Uroflowmetry was also performed and yielded normal results. On Day 17, the nephrostomy was removed, and a left ureteral stent was placed. The patient was then discharged on Day 25 from the onset of T1DM. Home therapy was based on a basal–bolus regimen with carbohydrate counting, nitrofurantoin 50 mg daily. Follow-up recommendations included performing a urological CT scan and a lumbosacral magnetic resonance imaging (MRI) in 6 weeks and periodic urine exams. Lumbosacral MRI was normal. Uro-CT showed normal kidneys and some mild ectasia in the right ureter with iodinated urine around the stent. Follow-up 24 hours urine collection showed no alteration in creatinine, uric acid, sodium, potassium, calcium, chloride, phosphate, magnesium, and citrate.

## 3. Discussion

We have reported the case of an almost 13-year-old boy diagnosed with T1DM and severe DKA which was complicated by an extremely rare and poorly described event: bilateral urinary lithiasis leading to urinary tract obstruction. We were unable to determine the exact composition of the stones, but the most likely form is calcium‐based stones (most often calcium oxalate and calcium phosphate). Marked hypocitraturia and hypomagnesuria combined with normal oxalate and calcium levels are most consistent with calcium stones. To date, only 4 cases of pediatric patients with new-onset T1DM and DKA at admission developed urinary stones [12, 17]. We found no other reported cases about bilateral urinary stones in the literature. Among the three children with newly diagnosed type 1 diabetes, a 24-h urine collection was performed in two cases. One child exhibited elevated urinary sodium levels, while the other showed reduced urinary citrate excretion [12]. Those pediatric patients with DKA had risk factors such as low citrate excretion, thus being consistent with the fact that metabolic disorders such as hypercalciuria, hyperuricosuria, hypocitraturia, cystinuria, and hyperoxaluria have been reported in 12%–50% of pediatric patients with urinary stones [13, 14, 18].

Another similar case, reported by Ushijima-Fuchino et al. [19], describes a 12-year-old adolescent with a new-onset T1DM and DKA who developed urinary stones. He had no known predisposing conditions. The patient experienced intermittent left lower abdominal pain 3 days after admission. Twelve hours after the onset of pain, the discomfort worsened and radiated to the back. A CT scan revealed left ureteric urinary stone (3 × 2 mm). The stone passed spontaneously with no further complication after the administration of 0.9% saline bolus, and a follow-up CT scan showed no residual urinary stones [19].

We started a comprehensive evaluation including urinalysis and laboratory blood tests that revealed no metabolic abnormalities, aside from those related to type 1 diabetes.

Children with DKA can experience various degrees of dehydration. Severe dehydration, hyperglycemia, and acidosis likely contributed to an environment favoring calcium crystallization, thereby leading to urinary stone formation [12].

On admission, clinical examination and laboratory tests confirmed dehydration; the DKA‐induced hyperglycemia‐related fluid loss contributed to urolithiasis. We also found that the patient had transitory hypocitraturia and hypomagnesuria. Citrates and magnesium increase the solubility of stone-forming calcium salts and inhibit the growth and aggregation of calcium oxalate crystals [20]. However, a definitive analysis of the stone composition could not be performed as the calculi were not retrieved during the procedures which represents a limitation of this report.

AKI is a recognized complication of DKA, and it is typically attributed to severe dehydration and renal hypoperfusion [9, 11]. Even though kidney stones associated with DKA are a rare but described event, bilateral stones leading to postrenal failure have never been described to our knowledge.

It is important to acknowledge that urolithiasis formation is typically a chronic process that develops over weeks to months or even years. Therefore, we could not definitively attribute the bilateral stones in this case to the acute metabolic derangements of DKA alone. It is plausible that the patient may have hadpre-existing lithiasis that had been asymptomatic or undetected and the profound dehydration, acidosis, and electrolyte disturbances associated with DKA acted as precipitating factors. These events may have worsened urinary concentration and crystal aggregation, promoted stone growth or migration and triggered bilateral obstruction leading to AKI. Furthermore, the presence of the intramural ureteral anatomical variant on the left side may have independently contributed to urinary stasis, potentially creating a nidus for stone formation that was acutely exacerbated by the metabolic derangements of DKA. The clinical course of the boy suggests that while standard DKA management protocols are effective in correcting metabolic abnormalities, clinicians should remain vigilant for secondary complications such as urinary obstruction, especially in patients presenting with atypical symptoms (e.g., lumbar pain and anuria). The multidisciplinary management, including prompt imaging, interventional radiology, and urological intervention, highlights the need for high index of suspicion and rapid response in such atypical presentations. This case may serve as a valuable teaching point, emphasizing the need for comprehensive evaluation and a collaborative approach among medical professionals to ensure optimal patient care.

## Data Availability

The data that support the findings of this study are available from the corresponding author upon reasonable request.
